# The genomes of three *Bradyrhizobium* sp. isolated from root nodules of *Lupinus albescens* grown in extremely poor soils display important genes for resistance to environmental stress

**DOI:** 10.1590/1678-4685-GMB-2017-0098

**Published:** 2018-05-17

**Authors:** Camille E. Granada, Luciano K. Vargas, Fernando Hayashi Sant’Anna, Eduardo Balsanelli, Valter Antonio de Baura, Fábio de Oliveira Pedrosa, Emanuel Maltempi de Souza, Tiago Falcon, Luciane M.P. Passaglia

**Affiliations:** 1 Universidade do Vale do Taquari Universidade do Vale do Taquari LajeadoRS Brazil Universidade do Vale do Taquari (UNIVATES), Lajeado, RS, Brazil; 2 Fundação Estadual de Pesquisa Agropecuária Fundação Estadual de Pesquisa Agropecuária Porto AlegreRS Brazil Fundação Estadual de Pesquisa Agropecuária, Porto Alegre, RS, Brazil; 3 Universidade Federal do Rio Grande do Sul Universidade Federal do Rio Grande do Sul Instituto de Biociências Departamento de Genética Porto AlegreRS Brazil Departamento de Genética, Instituto de Biociências, Universidade Federal do Rio Grande do Sul, Porto Alegre, RS, Brazil; 4 Universidade Federal do Paraná Universidade Federal do Paraná Departamento de Bioquímica e Biologia Molecular CuritibaPR Brazil Departamento de Bioquímica e Biologia Molecular, Universidade Federal do Paraná, Centro Politécnico, Curitiba, PR, Brazil; 5 Universidade de São Paulo Universidade de São Paulo Faculdade de Medicina de Ribeirão Preto Departamento de Genética Ribeirão PretoSP Brazil Departamento de Genética, Faculdade de Medicina de Ribeirão Preto, Universidade de São Paulo, Ribeirão Preto, SP, Brazil

**Keywords:** *Bradyrhizobium* sp., arenized areas, poor soils, resistant bacteria

## Abstract

*Lupinus albescens* is a resistant cover plant that establishes symbiotic relationships with bacteria belonging to the *Bradyrhizobium* genus. This symbiosis helps the development of these plants in adverse environmental conditions, such as the ones found in arenized areas of Southern Brazil. This work studied three *Bradyrhizobium* sp. (AS23, NAS80 and NAS96) isolated from *L. albescens* plants that grow in extremely poor soils (arenized areas and adjacent grasslands). The genomes of these three strains were sequenced in the Ion Torrent platform using the IonXpress library preparation kit, and presented a total number of bases of 1,230,460,823 for AS23, 1,320,104,022 for NAS80, and 1,236,105,093 for NAS96. The *genome comparison* with closest strains *Bradyrhizobium japonicum* USDA6 and *Bradyrhizobium diazoefficiens* USDA110 showed important variable *regions* (with less than 80% of similarity). Genes encoding for factors for resistance/tolerance to heavy metal, flagellar motility, response to osmotic and oxidative stresses, heat shock proteins (present only in the three sequenced genomes) could be responsible for the ability of these microorganisms to survive in inhospitable environments. Knowledge about these genomes will provide a foundation for future development of an inoculant bioproduct that should optimize the recovery of degraded soils using cover crops.

*Lupinus albescens* is a leguminous plant native of Uruguay, Paraguay, Northwestern Argentina, and Southern Brazil (Wolko *et al.*, 2011). This resistant cover plant presents the ability to grow in poor nutrient soils and is proven to be useful in strategies to recover degraded soils (Mihailovic *et al.*, 2008, Rovedder and Eltz, 2008). Its symbiosis with nitrogen fixing bacteria belonging to the *Bradyrhizobium* genus helps the growth and development of this plant in adverse environmental conditions, such as drought, salt excess, and heavy metal contamination (Jarabo-Lorenzo *et al.*, 2003, Fernández-Pascual *et al.*, 2007). *Bradyrhizobium* strains isolated from *L. albescens* plants are closely related with the *Bradyrhizobium japonicum* species, but these bacteria are somewhat different, as they are acid-tolerant and able to grow in soils with higher levels of free aluminum, opposed to acid-sensitive *B. japonicum* (Howieson *et al.*, 1998). Regions in southern Brazil present extremely poor soils that are prone to arenization, and the association between *L. albescens* plants and selected *Bradyrhizobium* species has been shown to be a good strategy for improving the potential of this plant to recover arenized and degraded sites (Rovedder and Eltz, 2008).

The genus *Bradyrhizobium* comprises rod-shaped Gram negative bacteria whose natural habitat is soil. This genus belongs to the phylum Proteobacteria, class Alphaproteobacteria, order Rhizobiales, family *Bradyrhizobiaceae*. The *Bradyrhizobium* strains studied in the present work were isolated from root nodules of *L. albescens* plants that grew in extremely poor soils prone to an intense degradation process (known as arenization) located in the southwestern region of the state Rio Grande do Sul, Brazil (Granada *et al.*, 2015). In previous work, Granada *et al.* (2015) demonstrated that the *Bradyrhizobium* strains isolated from root nodules of *L. albescens* were genetically different when the plants changed the environment (arenized areas in comparison with adjacent grassland). In the same work, a phylogenetic analysis (using *16S rRNA*, *dnaK*, *atpD*, *recA*, *glnII*, *rpoB*, *gyrB*, and *nodABZ* genes) encompassing all *Bradyrhizobium* reference species showed that isolates AS23 and NAS80 belong to a group where none of the reference species were allocated, while isolate NAS96 grouped with eight *Bradyrhizobium* reference species. In order to test the hypothesis that differences in the genomes of these three strains would reveal novel insights about microbial resistance to extreme poor soils, the genomes of isolates AS23 (isolated from a plant that grew in an arenized area), and NAS80 and NAS96 (isolated from plants that grew in adjacent grassland) were sequenced with the intention of finding genetic differences that could be connected to this resistance.

The three genomes were sequenced using the Ion Torrent platform and the IonXpress library preparation kit. The total number of bases, the quality score ≥ Q20 reads, and the average length of the reads are shown in Table 1. More than 84% of the bases had a quality score of ≥ Q20. To assemble the genomes, the MIRA software (v.4.0.2) (Chevreux *et al.*, 2004) was used, and general data of the assemblies were assessed by the software QUAST (v.4.3) (Gurevich *et al.*, 2013). The *de novo* assembled genomes, the number of total contigs, N50 contig length, and percentages of G + C content are also shown in Table 1. The sizes of the three genomes presented were similar to the genome size of the strains *B. japonicum* USDA 6 (9.2 Mb) and *B. diazoefficiens* USDA 110 (9.1 Mb). The assembled genomes were annotated in the online tool RAST (Aziz *et al.*, 2008, Overbeek *et al.*, 2014, Brettin *et al.*, 2015).

**Table 1:** Genome features of three *Bradyrhizobium* sp. isolates (AS23, NAS80, and NAS96) symbionts of *L. albescens* plants.

Features	AS23	NAS80	NAS96
Total number of bases	1,230,460,823	1,320,104,022	1,236,105,093
Q20 reads	11,381,012	12,690,871	11,319,891
Reads average length (bp)	108	104	109
Genomes size (bp)	8,705,820	9,235,468	8,928,139
Total contig number	430	533	451
N50 contig lengths (bp)	46,417	43,609	40,586
G + C content (%)	63.15	63.06	63.79
Number of subsytems	496	509	509
Number of tRNAs	47	61	50
Number of rRNAs (5S/16S/23S)	1/2/1	2/1/1	1/1/1
CDS	8540	9161	8390

Approximately 40% of all genes identified in the three sequenced genomes were classified into subsystems categories. The other genes were marked as unknown. The five most significant subsystems in these three genomes were: Amino Acids and Derivatives; Carbohydrates; Cofactors, Vitamins, Prosthetic Groups, Pigments; Membrane Transport and Fatty Acids, Lipids, and Isoprenoids (Figure 1). A comparison between the genomes of the three isolates with the genomes of the species *B. japonicum* USDA 6 and *Bradyrhizobium diazoefficiens* USDA 110 showed that the regions between positions 7,746 and 8,265 and 8,726 and 50 are the most conserved among the genomes of strains USDA 6 and USDA 110 (≥ 99%). These regions presented higher variability, with less than 80% of similarity, between the genomes of AS23, NAS80 and NAS96 and reference strains genomes (Figure 2, the genome of strain USDA6 was used as reference for the creation of this figure). The first region contains most of the genes related to nodulation and nitrogen fixation processes, especially the group of the *nod* (*A, C, U, Z)*, *nif (W, Q, O, Z, B, S, X, N, E)* and *nolN* genes, which presented less than 80% of similarity when compared with the same genes of the reference strain *B. japonicum* USDA 6. The second region contains mainly genes that coded for hypothetical proteins and mobile elements. A third region, between positions 3,090 and 3,385, presented low similarity between the genomes of our three isolates and the *B. diazoefficiens* USDA 110 genome in comparison with the *B. japonicum* USDA 6 genome. In this region, some genes for heat shock, as well as multidrug and heavy metals efflux transporters were identified.

**Figure 1:**
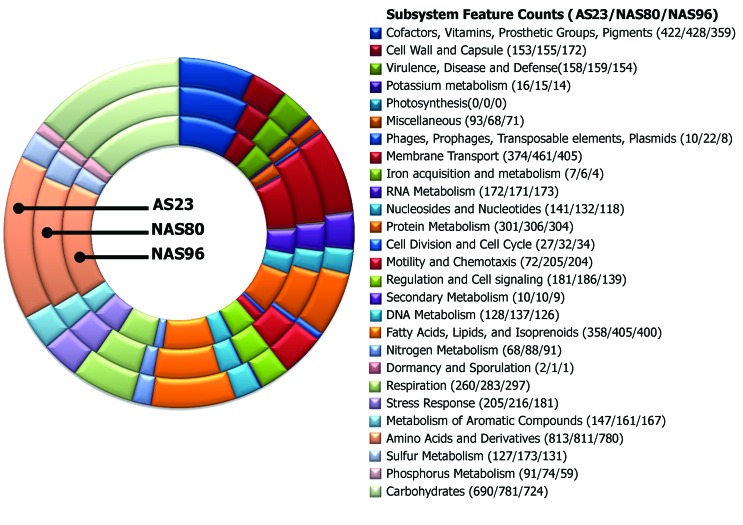
Subsystem category distribution and coverage of genes of the three *Bradyrhizobium* sp. isolates AS23, NAS80, and NAS96. In parenthesis are the number of genes in each subsystem for AS23, NAS80, and NAS96, respectively.

**Figure 2:**
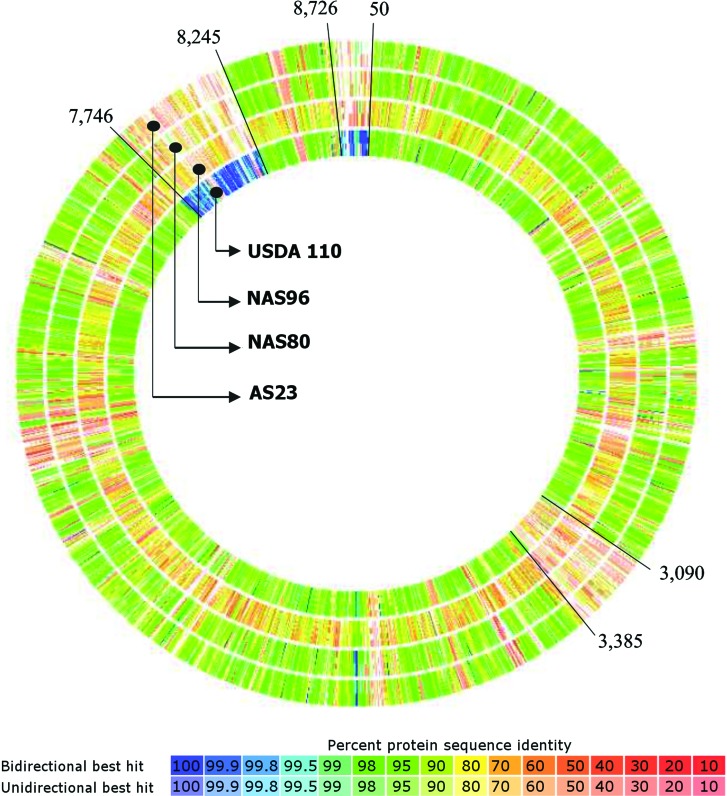
Circular heat map representing the genomes of *Bradyrhizobium* AS23, NAS80, NAS96, and *B. diazoefficiens* USDA 110 in comparison with the reference strain *B. japonicum* USDA 6 genome. Genome of the USDA 6 strain is not shown. The regions where the genes had less than 80% of similarity were considered significantly different.

The genomes of the three isolates presented 11,881 different genes, among which 4,072 genes (34.3%) were common to all genomes (Figure 3A). The number of genes unique to the NAS23 genome (1,423; 12%) was almost the same as the number of genes that was unique to the NAS80 genome (1,427; 12%), while the NAS96 genome presented the highest number of unique genes (2526; 21.3%). Few genes were common between the AS23 and NAS96 genomes (370 genes; 3.1%) and the NAS80 and NAS96 genomes (431 genes; 3.6%). The highest number of common genes was found among AS23 and NAS80 genomes (1,427; 12%). Adding the genome of the reference strain *B. japonicum* USDA 6 to the comparison (Figure 3B), genomes from AS23 and NAS80 resulted in a similar amount of genes shared with the reference genome (5,129 and 5,134, respectively), while the genome of isolate NAS96 shared 4,717 genes with the genome of USDA 6. Similarities among selected genes from *Bradyrhizobium* strains isolated from root nodules of *L. albescens* with the respective genes from *B. japonicum* species had already been observed by Stroschein *et al.* (2010) and Granada *et al.* (2015). However, both works suggested that those bacterial isolates probably constituted new *Bradyrhizobium* species. Thus, the present work provides results that are highly in support of this suggestion and adds that isolates AS23 and NAS80 probably belong to a new bradyrhizobial species, while the isolate NAS96 belongs to another new species.

**Figure 3:**
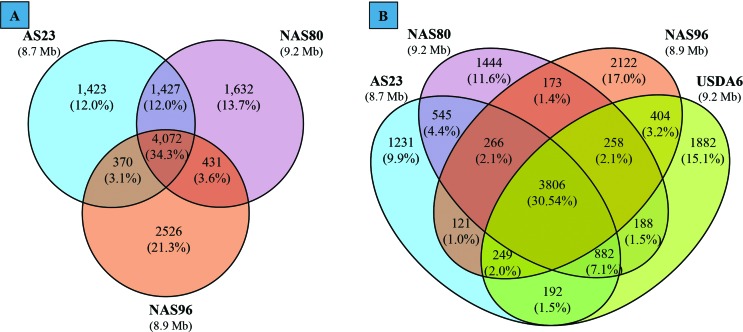
Venn diagrams comparing the genes identified in the genomes of *Bradyrhizobium* strains. (A) AS23, NAS80, and NAS96. (B) AS23, NAS80, NAS96, and USDA 6. Each genome is represented by a circle (A) or ellipse (B), and the numbers of shared and unique genes are shown by overlapping and non-overlapping parts, respectively. The proportion of total genes represented by each area of the diagram is shown in parentheses.

The reference strains *B. japonicum USDA 6* and *B. diazoefficiens* USDA 110 are symbionts with soybean plants, a crop plant that is typically cultivated in fertile soils. On the contrary, the strains analyzed in the present work were isolated from extremely poor soils, characterized by very low pH, poor clay and organic matter contents, and high levels of toxic aluminum and heavy metals (Granada *et al.*, 2013). Some genes that encode factors for resistance/tolerance to environmental stresses were present only in the three new genomes sequenced (from AS23, NAS80 and NAS96 isolates). Some of the genes were related to tolerance of heavy metals (cobalt, cadmium, zinc, arsenic, cooper, and chromium), flagellar motility (*flg*, *flh,* and *fli* families), response to osmotic and oxidative stresses (*aqua* and *sod* families), and heat shock (*grp* family). These genes could be responsible for the ability of these three microorganisms to survive in inhospitable environments, such as the ones found in southwestern Brazil (arenized areas and adjacent grasslands) from where they were isolated. These genes are good candidates for laboratory experiments with the aim of achieving deeper knowledge in the context of microbial resistance.

In summary, deeper knowledge of the three genomes described here will increase the understanding about bacterial adaptation to extremely poor soils and will elucidate the interaction mechanisms between these *Bradyrhizobium* isolates and *L. albescens*, evidencing a potential for future biotechnological application of an inoculant bioproduct to enhance the process of recovery of degraded soils using cover crops.

**Nucleotide sequence accession numbers.** This whole-genome shotgun project has been deposited at DDBJ/EMBL/GenBank under the accession numbers LGHM00000000 for AS23, LGHL00000000 for NAS80 and LGHK00000000for NAS96 (BioProject PRJNA289134; PRJNA289210 and PRJNA289232, respectively). The version described in this paper is the first version.
